# Research on coach-athlete relationship and team performance based on 3Cs theory: the chain mediating role of emotional intelligence and athletic engagement

**DOI:** 10.3389/fpsyg.2025.1587900

**Published:** 2025-06-18

**Authors:** Fangfang Pan, Wenjie Sui

**Affiliations:** Sports Institute, Huaqiao University, Xiamen, China

**Keywords:** coach-athlete relationship, team performance, emotional intelligence, athletic, 3Cs theory

## Abstract

In the Chinese cultural context, the coach-athlete relationship has a certain hierarchical structure, is dominated by coach-centered formal training, and its development is influenced by history and culture. Based on the 3Cs theory, this study constructs a mechanism model of how coach - athlete relationship impacts team performance. Using structural equation modeling, multilevel path analysis was conducted on 1,566 cross-sport athletes. By introducing two mediating variables, emotional intelligence and athletic engagement, the study explores the path of coach-athlete relationship on team performance. The research reveals that coach-athlete relationship exerts a notably positive influence on team performance (*β* = 0.556, *p* < 0.001), whose path of action showed a triple transduction dimension. Emotional intelligence has an independent mediating effect of 0.049 (*p* < 0.001). Athletic engagement shows an independent mediating effect of 0.094 (*p* < 0.001). The chained mediating effect amounts to 0.014 (*p* < 0.001). Results suggest that improving coach-athlete relationship, it can help to improve athletes’ emotional intelligence and engagement levels, thereby promoting team performance, which offers specific strategies and methods to boost team performance, holding great theoretical and practical significance.

## 1 Introduction

The cultivation mode of Chinese athletes is diversified, in which the coach-athlete relationship shows multiple attributes. Coaches integrate roles as leaders, strict mentors, and parental figures, playing a pivotal role in athletes’ training, education, and daily lives. The responsibility of coaches has extended beyond the traditional training field. They serve not only as instructors of professional skills but also as key guides in athletes’ career development. As the core carrier of teaching and learning, coach-athlete relationship influences not only athletes’ individual development such as self-awareness and self-esteem, but also directly impact training efficiency and competitive performance. A positive coach-athlete relationship enhances athletes’ self-efficacy, promoting more autonomous and internalized learning and training approaches, while also playing a proactive role in emotional regulation and mental health. Trust, respect, and effective communication play a key role in coach-athlete relationship. Conversely, trust deficits, lack of inclusivity, and communication barriers can undermine their collaborative foundation, thereby reducing the quality of training and competition (Ye et al., 2016).

Team performance (TP), as a core measure of team effectiveness, continues to receive attention in management, education, and sport (Chen et al., 2020). In competitive sports, it not only directly determines immediate competition performance and team reputation, but is also linked to economic benefits and brand value through fan economic effects (Funk et al., 2016), commercial sponsorship (Biscaia et al., 2013), and talent development. The composition of team performance is multidimensional. At the individual level, athletes’ physiological functions (Cormack et al., 2008), mental resilience (Gucciardi et al., 2015), and specific skills (Baker and Young, 2014) constitute foundational elements that affects team performance. At the organizational level, the training system (Impellizzeri et al., 2019), leadership behavior (Chelladurai and Saleh, 1980), and resource allocation (Sotiriadou and Shilbury, 2009) are considered as key variables affecting team performance. At the environmental level, policy support (Green and Houlihan, 2005), social capital (Welty Peachey et al., 2015), and economic investment (Downward and Riordan, 2007) constitute important moderators and act together on team performance. In short, team performance is not determined by a single variable, but is the result of a combination of multiple levels and factors.

When exploring team performance of sports, research has focused on two aspects. On one hand, numerous studies concentrate on internal factors of team such as athletes’ physical and mental state, the quality of daily training, peer support and coaches’ management style (Liu, 2024; Yan et al., 2024; Ramírez-Bravo et al., 2025). On the other hand, external factors of team such as policy environment, economic investment, and social capital have also received much attention (Molodchik et al., 2021). Although these factors are important, team performance is not solely dependent on this. Instead, it relies more on the presence of trust and communication between coaches and athletes (Jowett and Arthur, 2019). Previous studies have less frequently explored the effects of coach-athlete relationships on team performance. However, existing research has confirmed that compared to individual athlete abilities, the quality of coach-athlete relationship has a stronger predictive validity for team performance (Filho et al., 2014; Li, 2021). In competitive sport, teaching-learning interaction between coaches and athletes is central to team performance (Jowett, 2017), which has a dual mechanism. On one hand, it directly affects individual-level factors such as athletes’ self-perception, training effectiveness, and on-field performance. On the other hand, it has a systematic impact on performance by shaping team trust mechanisms and communication patterns. In addition, much exploration has focused on cognitive pathways such as goal setting, role identity, while neglecting the emotional transmission (Davis and Jowett, 2014).

Considering this, this study introduces emotional intelligence and combines the behavioral persistence characteristics of athletic engagement to construct a dual mediating model. The aim is to clarify the influencing relationships, mechanisms, and effects among variables. The research will reveal: how the coach-athlete relationship influences individual performance through emotional intelligence; how it transforms into sustained athletic engagement, which in turn affects team performance. This enriches the boundary conditions of impact mechanism of coach-athlete relationship on team performance and provides practical suggestions and theoretical references to enhance the development of sports teams.

## 2 Theoretical foundation and research hypothesis

### 2.1 Theoretical foundation

The 3Cs theory, used to explain coach-athlete relationships (Jowett and Lavalle, 2007), includes Closeness, Commitment and Complementarity. These three dimensions correspond to the emotional, cognitive, and behavioral components of relationship structures, respectively. Among them, Closeness refers to the emotional bond between coaches and athletes. Commitment reflects both parties’ willingness to establish and maintain the relationship. Complementarity manifests as effective interaction in their collaborative dynamics. The higher the coaches and athletes score on these three dimensions, the better the quality and effectiveness of the relationship will be.

Nonetheless, significant differences exist between China and Western countries in traditional culture, educational philosophies, and sports talent development systems. In the Chinese cultural context, coach-athlete relationship was developed in the embryonic form of the “master-apprentice” in traditional society. Influenced by Confucianism and traditional Chinese culture, coach-athlete relationship exhibits distinct ethical and hierarchical structures, embodied in concepts such as “respect teachers and value the way” and “collective interests above individual needs.” Coaches typically hold strong institutional and cultural authority, while athletes tend to maintain the relationship and facilitate teamwork through submission. Specifically, the coach-athlete relationship involves an ethical dimension of constructed identities, shaped implicitly by the logic of kinship and patriarchal structures, reinforcing a hierarchical order based on age and status. Generally speaking, coaches hold a dominant role while athletes remain subordinate, forming an unbalanced symbiosis (Yang and Jowett, 2013), sustained primarily by ethical kinship. As in the proverb, “Once a teacher, forever a father.” Studies show that trust, respect, and effective communication play pivotal roles in coach-athlete relationship, while trust deficits, lack of inclusivity, and communication barriers can undermine cooperation, which in turn degrades training and competition quality (Jowett and Palmer, 2010; Mchenry et al., 2020). In Chinese cultural contexts, athletes’ submissive behavior toward coaches stems not only from pressure but also reflects respect and trust. Such behavior facilitates unified tactical execution, reduces conflicts, clarifies role divisions, thus enhancing team cohesion (Ge et al., 2016). Athletes with higher submissiveness tend to demonstrate stronger execution ability and organizational identification (Ye et al., 2016). In summary, a positive coach-athlete relationship combined with appropriate submissive behaviors can effectively enhance team execution, serving as a critical guarantee for improving team performance.

Based on this, this article localizes the 3Cs theory. While retaining its three core dimensions, a new “submissiveness” dimension is added to authentically reflect the behavioral interaction patterns between coaches and athletes in the Chinese context, forming four dimensions: Closeness, Complementarity, Commitment, and Submissiveness.

### 2.2 Research hypothesis

#### 2.2.1 Coach-athlete relationship and team performance

Coach-athlete relationship (CAR) refers to a relatively stable psychological and behavioral bond formed during long-term training, competition, and communication interactions (Jowett, 2009). Whether the coach-athlete relationship is successful directly affects the growth rate of athletes, their competitive performance, and team performance (Nicholls et al., 2017). Optimizing the coach-athlete relationship and strengthening mutual understanding and support are the keys to improving team performance and achieving common goals. Based on this, the following hypothesis is proposed:

*H1*: The coach-athlete relationship can significantly and positively predict team performance.

#### 2.2.2 Mediating role of emotional intelligence

Emotional Intelligence (EI) is an individual’s ability to recognize, understand, manage his or her own emotions, and influence those of others. In a team, members with high EI can communicate effectively, manage conflicts, and boost team collaboration efficiency (Mysirlaki and Paraskeva, 2020; Zulfadil and Machasin, 2020). High level of EI can foster trust, team identity, and performance (Lyons and Schneider, 2005). Athletes with high EI are better at understanding coaches’ intentions, responding actively to challenges, reducing internal friction, and improving overall collaboration (Sun et al., 2024). Therefore, the study proposes the following hypothesis:

*H2*: Emotional intelligence mediates the influence of coach-athlete relationship on team performance.

#### 2.2.3 Mediating role of athletic engagement

Athletic engagement (AE) represents an enduring and positive cognitive and emotional experience toward sport behavior, grounded in individual psychology perception within sporting contexts. It amplifies dyadic interactions and directly influences team performance. A strong coach-athlete relationship fosters athletes’ psychological identification and training motivation, enabling them to maintain heightened focus and effort during training and competition (Wang and Tong, 2024). Accordingly, the following hypothesis is proposed:

*H3*: Athletic engagement mediates the influence of coach-athlete relationship on team performance.

#### 2.2.4 Chain mediating effect of emotional intelligence and athletic engagement

Emotional intelligence enhances the intensity and spontaneity of athletic engagement. Positive coaching-athlete relationship fosters a conducive training and competition environment, which promotes the development of athletes’ emotional intelligence. Athletes with high emotional intelligence are better at managing emotions and understanding needs of others (Murmu and Neelam, 2022), thereby stimulating athletic engagement. Moreover, athletes with high athletic engagement are more proactive in training and more committed in competition, leading to improved individual performance and ultimately boosting team performance. Accordingly, the following hypothesis is proposed:

*H4*: The coach-athlete relationship impacts team performance through a chained mediation of emotional intelligence and athletic engagement.

### 2.3 Research model

**Figure 1:**
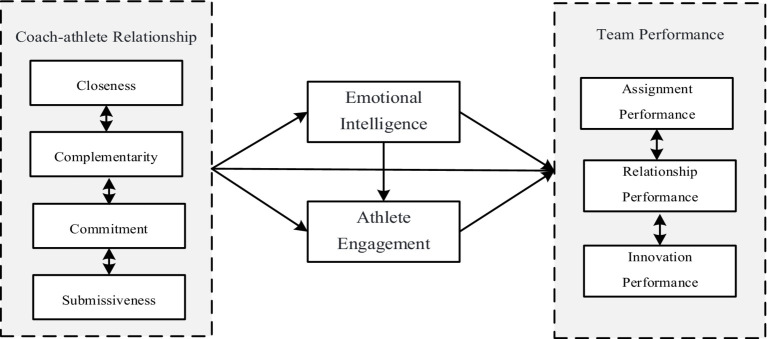
Theoretical model of the study.

Drawing on theoretical analysis and empirical validation, current study establishes a theoretical model examining the relationship between coach-athlete relationship and team performance. In this model, the coach-athlete relationship serves as the independent variable, while team performance is the dependent variable., emotional intelligence functions as the first mediator, and athletic engagement as the second mediator. This study aims to explore the relationship, mechanisms, and effects of coach-athlete relationship on team performance. Not only does this contribute to enriching and refining the theoretical framework related to coach-athlete relationship, but it also provides valuable insights and guidance for enhancing the performance and management of sports teams. The research model is as follows (see Figure 1).

## 3 Methodology

### 3.1 Ethics statement

This study adopts a questionnaire-based empirical research method and strictly adheres to academic norms and ethical requirements. All the information obtained is properly stored and processed anonymously. The entire research process is carried out meticulously in strict accordance with the guidelines and regulations established by Declaration of Helsinki. Meanwhile, this study has obtained approval from the relevant institutional review committee of Huaqiao University.

### 3.2 Participants

Based on the convenience sampling method, this study combines online and offline surveys to ensure the comprehensiveness and accuracy of data collection. The research participants broadly cover 24 sports teams from Fujian, Guangdong, Yunnan, Beijing, Shanghai, Shandong. Prior to distributing the questionnaires, the author actively contacted team leaders or head coaches, providing detailed explanations of purpose and process. Upon obtaining their full understanding and consent, a dual-track approach was adopted: on the one hand, questionnaire links were sent to team members via online survey platforms; on the other hand, the author personally visited some sports teams to conduct on-site surveys, engaging in face-to-face communication with team members and distributing paper questionnaires. To control for the impact of distribution methods on data, the study selected questionnaire formats based on team training schedules. Online questionnaires were used for sports with fragmented training times such as swimming, athletics, while offline questionnaires were administered in person for sports with centralized training such as basketball, dragon boat. Both formats contained identical content and were completed under the supervision of researchers or coaches to ensure data comparability and consistency.

### 3.3 Data collection

A total of 1,707 questionnaires were distributed in this study. After collecting the questionnaires, invalid ones such as those filled out repeatedly or with missing data were excluded, resulting in 1,566 valid questionnaires with a response rate of 91.74%. Of these, 1,098 were online questionnaires and 468 were offline questionnaires. The questionnaire survey broadly covered various sports including basketball, football, volleyball, dragon boat, dragon dance, lion dance, frisbee, etc. Specifically, the participants consist of 263 in athletics (16.79%), 318 in basketball (20.31%), 64 in table tennis (4.09%), 176 in badminton (11.24%), 206 in football (13.15%), 102 in tennis (6.51%), 106 in volleyball (6.77%), 122 in dragon boat (7.79%), 98 in dragon dance (6.26%), 42 in lion dance (2.68%), and 69 in frisbee (4.41%).

In terms of gender distribution, there were 1,053 male athletes, accounting for 67.24%, and 513 female athletes, accounting for 32.76%. Regarding age, most participants were aged between 18 and 25 years old. Additionally, the average training duration of the participants was 5.4 years. In terms of athlete grades, there were 99 national master sportsman, accounting for 6.32%; 792 national first-level athletes, accounting for 50.57%; 561 national second-level athletes, accounting for 35.82%; and 114 others, accounting for 7.28%.

### 3.4 Research instrument

#### 3.4.1 Dependent variable

In this study, the dependent variable is team performance, encompassing three dimensions: task performance, relational performance, and innovative performance, measured by 13 items (Yang and Sun, 2015; Gonzalez-Mule et al., 2014). Among them, task performance has 4 question items. Relationship performance has 5 items. Innovative performance has 4 items. Task performance refers to the completion of team tasks. This study uses athlete satisfaction as an evaluation indicator for task performance (Riemer and Chelladurai, 1998), encompassing two dimensions: satisfaction with individual performance and team performance. For example, I am satisfied with my personal performance on the team during training or competitions. My team can achieve shared goals. Relational performance involves behaviors beneficial to team building and development beyond training and competition, such as such as team identification, helping teammates, and sense of responsibility (Farh et al., 1997). For example, I am satisfied with my current teammates. Team members are willing to help each other solve problems in training or competitions. Veteran players assist new members in adapting to the team environment. Innovative performance reflects the team’s innovative efforts, such as proposing new training methods, tactical strategies, or improving existing processes (Li and Tang, 2022; Wahab et al., 2024). For example, team members actively contribute ideas for the team’s development. My team encourages the use of new methods and technologies to complete tasks. My team has strong adaptability to environmental changes. The questionnaire is scored on a five-point Likert scale, which athletes are asked to fill in according to actual situation. Higher scores indicate better team performance. The overall Cronbach’s *α* is 0.896, with a KMO of 0.926. Specifically, for task performance: KMO = 0.867, Cronbach’s α = 0.876; for relational performance: KMO = 0.808, Cronbach’s α = 0.830; and for innovative performance: KMO = 0.789, Cronbach’s α = 0.793.

#### 3.4.2 Independent variable

Coach-Athlete Relationship scale was designed with reference to Jowett (2005), which includes 14 items by adding the submissiveness to the original three dimensions of closeness, complementarity, and commitment. Specifically, closeness comprises 3 items, for instance, I like my coach. I trust my coach. Commitment includes 3 items, for instance, my relationship with my coach is close. I am willing to maintain a long-term cooperative relationship with my coach. Complementarity contains 3 items, for instance, I feel relaxed when my coach gives me guidance. I will respond positively when my coach guides me. Submissiveness includes 5 items, for instance, I am happy to accept my coach’s instructions and guidance. I am willing to accept my coach’s persuasion and suggestions. The questionnaire is scored on a five-point Likert scale, with five options ranging from “1 = Completely Disagree” to “Completely Agree,” with athletes instructed to respond based on actual experiences. Higher scores indicate a better-perceived coach-athlete relationship by athletes. The KMO value is 0.931, with a Cronbach’s *α* of 0.918.

#### 3.4.3 Mediating variables

Emotional Intelligence scale was compiled based on Wong and Law (2002), comprises four dimensions: self-emotional assessment, others’ emotional assessment, emotional expression and emotional regulation, with a total of seven items. The questionnaire is scored on a five-point Likert scale, with five options ranging from “1 = Completely Disagree” to “Completely Agree.” Higher scores indicate higher emotional intelligence among athletes. The KMO value is 0.897, with a Cronbach’s α of 0.869.

Athletic engagement scale was designed on the basis of Lonsdale et al. (2007), includes four dimensions: confidence, dedication, vigor, and enthusiasm. The questionnaire is scored on a five-point Likert scale, with five options ranging from “1 = Completely Disagree” to “Completely Agree.” The higher the score, the higher the commitment of athletes. The KMO value is 0.913 and the Cronbach’s α is 0.877.

### 3.5 Data processing

Based on descriptive statistics, correlation analysis, and multiple regression analysis using SPSS 27.0, this study further employs AMOS 27.0 and the PROCESS plugin to enhance the rigor and explanatory power of model analysis. AMOS is used for confirmatory factor analysis (CFA) and structural equation modeling (SEM) to test the construct validity of the theoretical model and causal pathways between variables. The PROCESS plugin conducts mediation effect tests based on regression analysis, generating confidence intervals and significance levels for mediating paths. This combination facilitates comprehensive validation of research hypotheses and strengthens the robustness of the findings.

## 4 Research results

### 4.1 Confirmatory factor analysis

In this study, confirmatory factor analysis was conducted using the maximum likelihood estimation method to validate the model. The results indicated the following model fit indices: χ^2^/df = 6.200, *p* < 0.001, RMSEA = 0.058, SRMR = 0.054, CFI = 0.896, TLI = 0.888, and IFI = 0.986. Although the χ^2^/df value in this study slightly exceeds the recommended threshold, this index is known to be sensitive to sample size. Larger samples may exaggerate *χ*^2^/df value (Kline, 2016). Additionally, as all other model fit indices fall within acceptable ranges, the overall model fit is still considered reasonable.

### 4.2 Reliability and validity

In this study, Cronbach’s *α* test is used for reliability. As shown in Table 1, the Cronbach’s α values are 0.918, 0.896, 0.869, and 0.877 respectively, and the KMO values range from 0.897 to 0.931, all exceeding the threshold of 0.7. The questionnaire reliability and validity are good.

**Table 1:** Reliability and validity test.

Variable	Dimension	Item	Std. estimate	Std. error	*P*	Cronbach’s *α*	AVE	KMO
Coach-athlete relationship	Closeness	CAR1	0.784	0.021	*P* < 0.001	0.918	0.613	0.931
		CAR2	0.844	0.022	*P* < 0.001			
		CAR3	0.885	–	–			
	Complementarity	CAR4	0.533	0.032	*P* < 0.001			
		CAR5	0.576	0.029	*P* < 0.001			
		CAR6	0.872	–	–			
	Commitment	CAR7	0.849	0.050	*P* < 0.001			
		CAR8	0.800	0.047	*P* < 0.001			
		CAR9	0.553	–	–			
	Submissiveness	CAR10	0.867	0.026	*P* < 0.001			
		CAR11	0.869	0.024	*P* < 0.001			
		CAR12	0.801	0.027	*P* < 0.001			
		CAR13	0.777	0.027	*P* < 0.001			
		CAR14	0.819	–	–			
Team performance	Assignment performance	TP1	0.823	–	–	0.896	0.558	0.926
		TP2	0.712	0.030	*P* < 0.001			
		TP3	0.731	0.033	*P* < 0.001			
		TP4	0.729	0.026	*P* < 0.001			
	Relational performance	TP5	0.780	–	–			
		TP6	0.902	0.028	*P* < 0.001			
		TP7	0.729	0.032	*P* < 0.001			
		TP8	0.726	0.034	*P* < 0.001			
		TP9	0.719	0.034	*P* < 0.001			
	Innovation performance	TP10	0.659	0.037	*P* < 0.001			
		TP11	0.620	0.037	*P* < 0.001			
		TP12	0.739	0.034	*P* < 0.001			
		TP13	0.799	–	–			
Emotional intelligence	Single dimensionality	EI1	0.567	–	–	0.869	0.506	0.897
		EI2	0.588	0.053	*P* < 0.001			
		EI3	0.843	0.065	*P* < 0.001			
		EI4	0.860	0.067	*P* < 0.001			
		EI5	0.584	0.067	*P* < 0.001			
		EI6	0.797	0.065	*P* < 0.001			
		EI7	0.667	0.062	*P* < 0.001			
Athletic engagement	Single dimensionality	AE1	0.628	–	–	0.877	0.514	0.913
		AE2	0.590	0.052	*P* < 0.001			
		AE3	0.772	0.053	*P* < 0.001			
		AE4	0.724	0.059	*P* < 0.001			
		AE5	0.702	0.053	*P* < 0.001			
		AE6	0.825	0.054	*P* < 0.001			
		AE7	0.749	0.054	*P* < 0.001			

Furthermore, the Average Variance Extracted (AVE) values are located in the range of 0.506–0.613, all of which are >0.5. The factor loading coefficients range from 0.533 to 0.902 with *p* < 0.001 and all exceeding 0.5, which suggests that the items of the scales correspond well with their respective dimensions, demonstrating good convergent validity.

### 4.3 Descriptive statistics

Statistical analysis using SPSS 27.0 reveals that four scales all significantly exceed the theoretical median of 3, with relatively low data dispersion (see Table 2). In terms of coach-athlete relationship, the mean is 3.989 with a standard deviation of 0.527, indicating an overall strong relationship with low dispersion and harmonious interactions among most. High scores in closeness, submissiveness, and complementarity reflect good emotional bonds, high cooperation, and effective complementary patterns. Commitment scores are relatively lower, showing variations among athletes, potentially linked to individual personality and values. For team performance, the mean is 3.895 with a standard deviation of 0.604, indicating a moderately high performance with low dispersion and stable performance. Relational performance significantly surpasses task and innovative performance, suggesting athletes excel in interpersonal collaboration but need improvement in task execution and innovation. Emotional intelligence has a mean of 3.538 with a standard deviation of 0.650, indicating a moderate level with variations among athletes. The mean athletic engagement of 3.60 suggests varying degrees of athlete commitment. Combined with the mean emotional intelligence, emotional regulation abilities may limit athletes’ focus, or it could be related to their level of passion and goals.

**Table 2:** Descriptive statistic.

Variable	M	SD	Variable	M	SD
Closeness	4.031	0.623	Athletic engagement	3.604	0.702
Complementarity	4.019	0.637	Assignment performance	3.819	0.721
Commitment	3.812	0.717	Relational performance	4.000	0.733
Submissiveness	4.051	0.607	Innovative performance	3.838	0.735
Coach-athlete relationship	3.989	0.527	Team performance	3.895	0.604
Emotional intelligence	3.538	0.650	–	–	–

### 4.4 Multiple regression analysis

The correlation matrix is presented in Table 3. Based on the correlation analysis results, all variables are directly correlated with each other. Additionally, the square roots of AVE for each pair of variables are all greater than the correlation coefficients between them. The Variance Inflation Factor (VIF) values are all below 10, indicating a negligible likelihood of multicollinearity in the model. Therefore, it is suitable to proceed with further analysis.

**Table 3:** Correlation analysis.

Variable	1	2	3	4
1. Coach-athlete relationship	(0.783)			
2. Emotional intelligence	0.272^**^	(0.747)		
3. Athletic engagement	0.493^**^	0.350^**^	(0.711)	
4. Team performance	0.623^**^	0.356^**^	0.486^**^	(0.710)

*p < 0.05; **p < 0.01; ***p < 0.001.

Subsequent regression analysis results are summarized in Table 4. Each factor significantly impacts assignment performance, relational performance, and innovative performance, albeit with varying degrees of influence and specific manifestations. Taking the three dimensions of team performance as dependent variables and coach-athlete relationship as the independent variable, hierarchical regression analysis was conducted on emotional intelligence and athletic engagement to test mediating roles, based on the main effect. Firstly, Models 1, 4, and 7 demonstrate significant main effects, indicating a significant positive impact of coach-athlete relationship on team performance. Secondly, Models 2, 5, and 8, incorporating the first mediator on top of the independent variable, show that emotional intelligence plays a mediating role. Furthermore, Models 3, 6, and 9, adding the second mediator, still show significant effects, indicating that athletic engagement also mediates between the independent and dependent variables. In other words, emotional intelligence and athletic engagement jointly exhibit a chain-mediating effect in the influence pathway of coach-athlete relationship on team performance.

**Table 4:** Multiple regression analysis.

Independent variable	Std. estimate	Std. error	T	Std. estimate	Std. error	T	Std. estimate	Std. error	T	VIF
Dependent variable: assignment performance										
	Model 1			Model 2			Model 3			
Closeness	0.193^***^	0.048	4.641	0.236^***^	0.046	5.883	0.238^***^	0.045	6.111	3.874
Complementarity	0.250^***^	0.030	9.297	0.198^***^	0.030	7.566	0.166^***^	0.029	6.478	1.677
Commitment	0.131^***^	0.026	5.080	0.140^***^	0.025	5.645	0.091^***^	0.025	3.665	1.555
Submissiveness	0.088^*^	0.049	2.125	0.007	0.048	0.180	−0.035	0.047	−0.871	3.995
Emotional intelligence				0.251^***^	0.024	11.684	0.200^***^	0.024	9.230	1.190
Athletic engagement							0.222^***^	0.024	9.339	1.441
R^2^	0.296			0.353			0.387			
Adjusted R^2^	0.295			0.351			0.385			
F	164.349			170.200			164.207			

Independent variable	Std. estimate	Std. error	T	Std. estimate	Std. error	T	Std. estimate	Std. error	T	VIF
Dependent variable: relational performance										
	Model 4			Model 5			Model 6			
Closeness	0.154^***^	0.050	3.639	0.168^***^	0.050	3.975	0.170^***^	0.049	4.031	3.874
Complementarity	0.151^***^	0.031	5.522	0.133^***^	0.032	4.836	0.117^***^	0.032	4.242	1.677
Commitment	0.181^***^	0.027	6.884	0.184^***^	0.027	7.020	0.159^***^	0.027	5.952	1.555
Submissiveness	0.155^***^	0.051	3.660	0.128^**^	0.052	2.988	0.106^*^	0.052	2.487	3.995
Emotional intelligence				0.084^***^	0.026	3.693	0.058^*^	0.026	2.468	1.190
Athletic engagement							0.113^***^	0.027	4.416	1.441
R^2^	0.273			0.279			0.288			
Adjusted R^2^	0.271			0.277			0.286			
F	146.651			120.998			105.278			

Independent variable	Std. estimate	Std. error	T	Std. estimate	Std. error	T	Std. estimate	Std. error	T	VIF
Dependent variable: innovative performance										
	Model 7			Model 8			Model 9			
Closeness	0.011	0.051	0.261	0.045	0.050	1.056	0.046	0.049	1.107	3.874
Complementarity	0.202^***^	0.032	7.278	0.161^***^	0.032	5.867	0.141^***^	0.032	5.137	1.677
Commitment	0.179^***^	0.028	6.704	0.186^***^	0.027	7.119	0.154^***^	0.027	5.823	1.555
Submissiveness	0.218^***^	0.052	5.075	0.155^***^	0.052	3.631	0.128^**^	0.051	3.010	3.995
Emotional intelligence				0.196^***^	0.026	8.672	0.163^***^	0.026	7.042	1.190
Athletic engagement							0.143^***^	0.027	5.597	1.441
R^2^	0.249			0.283			0.298			
Adjusted R^2^	0.247			0.281			0.295			
F	129.280			123.380			110.036			

*p < 0.05; **p < 0.01; ***p < 0.001.

The study employed Model 6 of PROCESS in SPSS software, utilizing Bootstrap, with a put-back repetition of 5,000 times of sampling, to test whether the mediating effect is significant according to whether the 95% confidence interval contains 0 or not. If the confidence interval did not contain 0, it indicated a significant mediating effect. The results are presented in Table 5, where all 95% confidence intervals exclude 0, indicating significant mediating effects. Specifically, the direct effect (CAR → TP) was 0.556 (*p* < 0.001) with a standard error of 0.025 and a 95% confidence interval ranging from 0.508 to 0.605. The total indirect effect (CAR → EI → AE → TP) was 0.157 (*p* < 0.001) with a standard error of 0.017 and a 95% confidence interval of 0.124–0.192. The total effect (direct effect + indirect effect) was 0.713 (*p* < 0.001) with a standard error of 0.023 and a 95% confidence interval of 0.669–0.758.

**Table 5:** Mediated effect pathways.

Intermediary path	Std. estimate	Std. error	95% confidence interval		Effect ratio
			LLCL	ULCL	
CAR→EI → TP	0.049^***^	0.011	0.027	0.071	6.87%
CAR→AE → TP	0.094^***^	0.017	0.620	0.129	13.18%
CAR→EI → AE → TP	0.014^***^	0.004	0.008	0.022	1.96%
Total indirect effect	0.157^***^	0.017	0.124	0.192	22.02%
Direct effect	0.556^***^	0.025	0.508	0.605	77.98%
Total effect	0.713^***^	0.023	0.669	0.758	100%

*p < 0.05; **p < 0.01; ***p < 0.001.

### 4.5 Structural equation test and analysis

**Figure 2:**
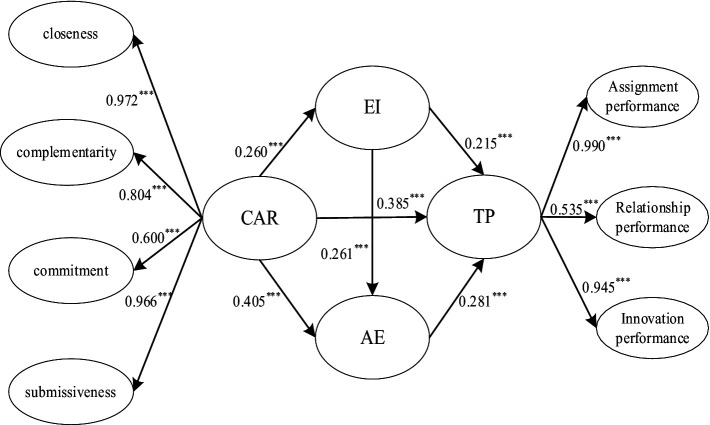
Hypothetical path diagram.

Due to the complexity of multiple mediation models, which involve numerous variables and intricate pathways, structural equation modeling (SEM) is suitable for analysis. In this study, dimensions of coach-athlete relationship and team performance were treated as their respective observed indicators, while seven items for emotional intelligence and athletic engagement served as their corresponding observed indicators, forming separate measurement models. Analysis of models revealed that all indicator variables significantly loaded onto their respective latent variables, indicating the validity of the measurement instruments and the rationality of the item parceling strategy (see Figure 2).

The hypothesis testing encompasses two components: direct and indirect effects. The standardized coefficient values in Table 6 indicate that the coach-athlete relationship has a significant impact on team performance, with a point estimate of 0.385, confirming Hypothesis H1. Furthermore, the coach-athlete relationship significantly influences emotional intelligence (*β* = 0.260, *p* < 0.001), and emotional intelligence, in turn, significantly affects team performance (*β* = 0.215, *p* < 0.001). Thus, emotional intelligence mediates between two, validating Hypothesis H2. The coach-athlete relationship also exhibits a significant influence on athletic engagement (*β* = 0.405, *p* < 0.001), which positively impacts team performance (*β* = 0.281, *p* < 0.001). Athletic engagement mediates between the two, confirming Hypothesis H3. Additionally, emotional intelligence positively influences athletic engagement, with a point estimate of 0.261, supporting Hypothesis H4.

**Table 6:** Direct, indirect, and total effect.

Path	Estimates	S. E.	Bootstrap 5,000 times 95% CI				Effect ratio
			Percentile		Bias-corrected		
			Lower	Upper	Lower	Upper	
Direct effect							
CAR→TP	0.385^***^	0.042	0.304	0.466	0.302	0.464	67.19%
CAR→EI	0.260^***^	0.039	0.184	0.337	0.186	0.342	100%
CAR→AE	0.405^***^	0.032	0.341	0.465	0.340	0.465	85.62%
EI → TP	0.215^***^	0.040	0.128	0.300	0.128	0.300	74.65%
AE → TP	0.281^***^	0.037	0.206	0.356	0.204	0.354	100%
EI → AE	0.261^***^	0.030	0.201	0.320	0.201	0.320	100%
Indirect effect							
CAR→TP	0.188^***^	0.020	0.151	0.227	0.152	0.229	32.81%
CAR→AE	0.068^***^	0.014	0.043	0.096	0.044	0.0980	14.38%
EI → TP	0.073^***^	0.013	0.050	0.100	0.051	0.101	25.35%
Total effect							
CAR→TP	0.573^***^	0.034	0.506	0.604	0.505	0.639	100%
CAR→EI	0.260^***^	0.039	0.184	0.337	0.186	0.342	100%
CAR→AE	0.473^***^	0.032	0.409	0.533	0.409	0.533	100%
EI → TP	0.288^***^	0.038	0.212	0.363	0.211	0.362	100%
AE → TP	0.281^***^	0.037	0.206	0.356	0.204	0.354	100%
EI → AE	0.261^***^	0.030	0.201	0.320	0.201	0.320	100%

*p < 0.05; **p < 0.01; ***p < 0.001.

## 5 Discussion

The study centers on the coach-athlete relationship and team performance, revealing a significant correlation between them. Emotional intelligence and athletic engagement serve as mediating factors. Furthermore, the chain mediation effect of emotional intelligence and athletic engagement was verified.

### 5.1 Coach-athlete relationship and team performance

This study reveals a statistically significant correlation between coach-athlete relationship and team performance (*β* = 0.556, *p* < 0.001). Favorable coach-athlete relationships foster a positive team atmosphere and enhance team cohesion, ultimately boosting team performance. Specifically, closeness (*β* = 0.143^***^), commitment (*β* = 0.170^***^), complementarity (*β* = 0.225^***^) and submissiveness (*β* = 0.185^***^) all positively affected team performance, with the most significant effect of complementarity. Complementarity reflects the coordination between coaches and athletes in behavioral styles and role functions. As a key driver of performance improvement, it directly impacts team efficiency and training outcomes. In Chinese culture, individuals tend to integrate personal values with collective goals, which is beneficial in further reinforcing the role of complementarity in improving team efficiency and training outcomes. Commitment manifests as psychological contracts and goal consensus, with its core value lying in establishing long-term stable partnerships, enhancing responsibility and team loyalty, rather than merely pursuing short-term coordination efficiency. While submissiveness is a positive factor, its effectiveness depends on submissiveness rooted in mutual recognition and interaction. Unidirectional submissiveness without recognition and interaction is difficult to translate into high-quality performance. Therefore, during training, it is essential to fully consider individual differences, reasonably adjust training loads, and prioritize athletes’ autonomy. The relatively low direct effect of closeness on performance suggests that in a performance-oriented competitive environment, affective connections play mainly a supportive role such as moderating and buffering effects. In sum, performance improvement is not solely dependent on either authority or emotion, but rather an integration of behavioral collaboration and psychological commitment based on mutual trust. A stable coach-athlete relationship is crucial for ensuring training execution and alignment with collective goals (Si et al., 2011; Weinberg and Gould, 2015). Therefore, coaches should act not only as mentors but also as trusted allies to athletes, which requires continuous adjustment of management approaches, prioritizing communication and collaboration to foster positive relationships and enhance team performance.

### 5.2 Mediating role of emotional intelligence

The coach-athlete relationship not only directly influences team performance but also indirectly through emotional intelligence, with a combined effect of 0.049^***^. A favorable coach-athlete relationship creates a positive feedback environment that promotes emotional intelligence in athletes (Jowett et al., 2024). When coaches and athletes establish strong emotional connections, athletes are more willing to reveal their true emotions. Coaches can detect psychological changes and adjust training strategies flexibly accordingly. When athletes recognize coaching authority and voluntarily comply with guidance, they can effectively reconcile personal emotions with team needs based on clear collective goals. In addition, emotional intelligence is known to be instrumental in team performance enhancement (Tamminen et al., 2019). Emotional intelligence, as a cultivable ability, aids athletes in recognizing, regulating, and managing emotions, enabling them to remain calm and maintain focus under pressure (Lee and Wong, 2017). Meanwhile, athletes with high emotional intelligence are better at recognizing and understanding the emotions of their teammates, resolving interpersonal conflicts in a timely manner, promoting emotional coordination and collaborative atmosphere within the team, finally enhancing overall team performance (Zulfadil and Machasin, 2020). Improving emotional intelligence takes time and practice, which can be facilitated through emotion management and psychological counseling between coaches and athletes. Team managers should emphasize emotional intelligence in team development, incorporating it into team-building activities, so as to optimize coach-athlete relationship and boosts team performance.

### 5.3 Mediating role of athletic engagement

Coach-athlete relationship not only directly affects team performance but also indirectly affects it through athletic engagement, with a total effect size of 0.094^***^. When athletes feel respected, understood, and supported by their coaches, they develop psychological safety, which strengthens their goal orientation and training motivation. A secure relational environment helps mitigate athletes’ fear of failure, empowering them to embrace trial-and-error and pursue breakthroughs. At the same time, a good coach-athlete relationship improves athletes’ intrinsic acceptance of training goals and promotes athletic engagement (Gu et al., 2023). Additionally, athletic engagement significantly enhances team performance. High levels of engagement typically manifest as stronger focus, greater intensity of effort, and longer sustained engagement. It accelerates athletes’ skill internalization and automation, improving the stability of technical movements and the accuracy of tactical execution. However, athletic engagement varies individually and is influenced by multiple factors, such as personal interest, career planning, and family support. Excessive engagement may lead to emotional exhaustion. Coaches should design training programs reasonably such as referring to the SMART principle, so that the athletes’ commitment level is in a reasonable interval to optimize team performance (Lu et al., 2022).

### 5.4 Chain mediation effect

This study confirms the chained mediation effect of emotional intelligence and athletic engagement (*β* = 0.014, *p* < 0.001). Specifically, coaches’ behaviors first enhance athletes’ emotional intelligence, which in turn increases athletic engagement, ultimately improving team performance. This aligns with the cognition-emotion-behavior model, where external stimuli (coach support) initially alter individual emotional regulation capabilities, subsequently driving consistent behavioral engagement. Athletes with high emotional intelligence train and compete more positively, enhancing their athletic engagement, thereby fostering rapport with teammates, and driving team performance. Management should prioritize fostering a positive team environment by starting with improving coach-athlete relationships, establishing support systems, regularly assessing and enhancing athletes’ emotional intelligence and athletic engagement, and strengthening team culture to achieve sustained growth in team performance.

## 6 Contribution

This study, grounded in the 3Cs theory, analyzes coach-athlete relationships and explores how they influence trust and collaboration within teams, ultimately enhancing team performance. It emphasizes how coach-athlete relationships translate into team dynamics and outcomes. Through empirical research, it provides crucial insights for professional team management. Additionally, by examining the mediating roles of emotional intelligence and athletic engagement, this study offers guidance to coaches on how to optimize collaboration in team practices, furnishing a significant management framework and empirical reference for team management and coaching practices.

## 7 Limitation

This study proposes that emotional intelligence and athletic engagement serve as partial mediating variables between coach-athlete relationships and team performance. However, it is undeniable that other unidentified mediating or moderating variables may also exist, warranting further exploration in future research. The study used cross-sectional data, limiting inferences about causality. Future studies could use longitudinal data to increase explainability. Variable measurement primarily relies on self-reports, which may introduce social desirability bias (Noroozi et al., 2024). Future studies could incorporate other-rated assessments, interviews, or behavioral observations to enhance data quality. Although no serious common method bias (CMV) problems were found in this article, future studies could introduce procedural or statistical control methods, such as the Harman one-way test, to enhance the reliability and validity of the results. In addition, the study focuses on specific variables and pathways and is limited by quantitative thinking. In the future, a combination of quantitative and qualitative methods can be used to conduct comparative studies in different sports and cultural contexts to enhance the breadth and applicability of the study (Creemers and Kyriakides, 2007).

## Data Availability

The original contributions presented in the study are included in the article/supplementary material, further inquiries can be directed to the corresponding author.
